# Collaborative Research Project: Developing and Testing a Robot-Assisted Intervention for Children With Autism

**DOI:** 10.3389/frobt.2020.00037

**Published:** 2020-03-31

**Authors:** Viviane Kostrubiec, Jeanne Kruck

**Affiliations:** ^1^Centre d'Etudes et de Recherches en Psychopathologie et Psychologie de la Santé (CERPPS), Université de Toulouse, UT2J, Toulouse, France; ^2^Université de Toulouse, UT3, Toulouse, France

**Keywords:** social robotics, social skills, evidence-based practices, robot acceptance, applied analysis of behavior

## Abstract

The present work is a collaborative research aimed at testing the effectiveness of the robot-assisted intervention administered in real clinical settings by real educators. Social robots dedicated to assisting persons with autism spectrum disorder (ASD) are rarely used in clinics. In a collaborative effort to bridge the gap between innovation in research and clinical practice, a team of engineers, clinicians and researchers working in the field of psychology developed and tested a robot-assisted educational intervention for children with low-functioning ASD (*N* = 20) A total of 14 lessons targeting requesting and turn-taking were elaborated, based on the Pivotal Training Method and principles of Applied Analysis of Behavior. Results showed that sensory rewards provided by the robot elicited more positive reactions than verbal praises from humans. The robot was of greatest benefit to children with a low level of disability. The educators were quite enthusiastic about children's progress in learning basic psychosocial skills from interactions with the robot. The robot nonetheless failed to act as a social mediator, as more prosocial behaviors were observed in the control condition, where instead of interacting with the robot children played with a ball. We discuss how to program robots to the distinct needs of individuals with ASD, how to harness robots' likability in order to enhance social skill learning, and how to arrive at a consensus about the standards of excellence that need to be met in interdisciplinary co-creation research. Our intuition is that robotic assistance, obviously judged as to be positive by educators, may contribute to the dissemination of innovative evidence-based practice for individuals with ASD.

## 1. Introduction

There is a growing recognition of the innovation-to-practice gap arisen in social robotics (Fernaeus et al., 2010; Pennisi et al., 2016; Walters, 2018; Ismail et al., 2019), a field dedicated to developing robots to assist persons with special needs. To date, few social robots have gone beyond the prototype stage, or else are only deployed for research purposes (Wagenmakers, 2016). Their sale volume is still low (6,423 units in 2017), compared with that of domestic help robots (6.1 million units in 2017) (IFR, 2018). Kim et al. (2012) (see also Cabibihan et al., 2013; Pennisi et al., 2016) ascribed these difficulties to the lack of collaboration between researchers and end-users. Too long, research effort focused on the technological features of newly engineered robots (e.g., Kozima et al., 2007; Robins et al., 2009), not taking into account the specific needs of end-users. End-users do not evaluate a technical innovation, however outstanding it may be (Payne, 2015). Rather, they evaluate its added value relative to existing alternatives and its accordance to work routines (Joachim et al., 2018).

The hard earned lesson now is that to overcome the innovation-to-practice gap, close collaboration between engineers, researchers, caregivers and management team is needed. The collaboration may take the form of a participatory, pragmatic, or *collaborative approach*, where all the stakeholders work hand in hand to co-create tools best fitting the needs of end-users (Schwartz and Lellouch, 1967; March et al., 2005; Zwarenstein et al., 2008; Marchand et al., 2011; Forman et al., 2013; Bauer et al., 2015). In this emerging framework, having recently gained impetus from the paper by Balas and Boren (2000), researcher does not solely ask whether a new tool works when used in optimal laboratory conditions. Rather, he evaluates whether the tool works when used in real-life clinical settings, without highly-qualified staff, a homogenous group of patients, or tight experimental control (Cargo and Mercer, 2008; Zwarenstein et al., 2008; Brownson et al., 2012). The tool's acceptance is assessed by a questionnaire and implementation failures and context reported as a result on its own (Stahmer et al., 2015). We exploit here the collaborative approach to co-create and test socially assistive robot during an educational intervention dedicated to children with *autism spectrum disorder* (ASD).

### 1.1. Robots and ASD

ASD is an early-onset, pervasive developmental disorder that manifests itself in anomalies in social communication and interaction, together with abnormal restricted and/or repetitive patterns of behavior and interests (Lord et al., 1994; DSM 5, 2013). For instance, children with ASD avoid physical contact, do not orient toward humans, do not point to communicate, do not express enjoyment or interest, and may spend hours at lining up toys or flipping objects (Rutter et al., 2003). As ASD is incurable, some persons with this disorder require costly and intensive lifetime care, support and treatment, motivating the development of social robots to assist them and their caregivers.

The arising of social robots dedicated to ASD can be traced back to the seminal study by Emanuel and Weir (1976) (see also Howe, 1983), where a computer-controlled electrotechnical device, a turtle-like robot (LOGO) moving on wheels around the floor, was used as a remedial tool for an ASD boy. It was not until the late 1990s that multiple laboratories adopted this topic for research (see Werry and Dautenhahn, 1999; Diehl et al., 2012; Begum et al., 2016; Ismail et al., 2019; for reviews).

To date, nearly 30 robots were tested as remedial tools for ASD [e.g., : Labo-1 (Werry et al., 2001); Muu (Miyamoto et al., 2005), Robota (Billard et al., 2007), FACE (Pioggia et al., 2007), Keepon (Kozima et al., 2007), Aibo (Francois et al., 2009), IROMEC (Iacono et al., 2011), Charlie (Boccanfuso and O'Kane, 2011), NAO (Shamsuddin et al., 2012), Flobi (Damm et al., 2013); GIPY-1 (Giannopulu, 2013), Pleo (Kim et al., 2013), KASPAR (Wainer et al., 2014), Darwin-OP (Peng et al., 2014), Pabi (Dickstein-Fischer and Fischer, 2014), Zeno (Salvador et al., 2015), Jibo (Guizzo, 2015), Probo (Simut et al., 2016), Maria (Valadao et al., 2016), Sphero (Golestan et al., 2017), CARO (Yun et al., 2017), KiliRo (Bharatharaj et al., 2018), MINA (Ghorbandaei Pour et al., 2018), QTrobot (Costa et al., 2018), Milo (Chalmers, 2018), Leo (She et al., 2018), Daisy (Pliasa and Fachantidis, 2019), SAM (Lebersfeld et al., 2019), SPRITE (Clabaugh et al., 2019), Actroid-F (Yoshikawa et al., 2019) etc.].

The key hypothesis behind this endeavor states that social robots can maybe overcome some of the motivational and sensory barriers encountered by individuals with ASD when they interact with humans partners (Dautenhahn, 1999). In contrast to their typically developing peers, for whom social interactions are inherently rewarding, children with ASD exhibit only weak activation of the brain's reward system in response to social reinforcement (Chevallier et al., 2012; Delmonte et al., 2012; Watson et al., 2015). *Social Motivation Theory of ASD*, Chevallier et al. (2012) argued that ASD children neither seek out nor seek to maintain relations with human partners, showing instead a preference for nonhuman and often mechanic stimuli (Watson et al., 2015).

In addition to these motivational issues, sensory processing of persons with ASD is abnormal: they are often intolerant of complex multimodal stimuli (Bogdashina, 2010, 2012), display detail-focused perception (Happé and Frith, 2006), and sensory sensitivities or aversions (Bogdashina, 2010), with intense social anxiety (Spain et al., 2018). According to the *Weak Central Coherence theory* (Happé et al., 2001) and *Enhanced Perceptual Functioning model* (Mottron et al., 2006), the perceptual processing of ASD persons is biased toward local features: these children are incapable of integrating the variety of individual pieces of information into global patterns. *Intense World Theory of Autism* (Markram, 2007) sugested that these persons suffer from excessive neuronal information processing causing informational overload and abnormal levels of anxiety, which they seek to reduce with stereotypical and repetitive behaviors (Rodgers et al., 2012).

Given these characteristics of ASD, it seems useful to examine whether a social robot, with its motivational appeal, behavioral repetitiveness, simplified appearance and lack of social judgment, may be more appealing to individuals with ASD than real humans. Therefore, in line with Social Motivation Theory of ASD (Chevallier et al., 2012) our first working hypothesis (Hypothesis 1) is that children with ASD should positively react to sensory rewards delivered by a robot, by manifesting their interest and satisfaction when these stimuli are provided. In line with Intense World Theory of Autism (Markram, 2007), we also expect a reduction of anxiety-related undesirable behaviors (e.g., stereotypes, screams, auto-aggressions, etc.) in the presence of the robot (Hypothesis 2).

Yet, the key hope behind social robotics for ASD is that robots act as *social mediators*: they mediate, that is, promote or “catalyze” a cascade of so-called prosocial behaviors directed toward humans: eye or head orienting, physical contact, pointing to shared interest etc. (Dautenhahn, 2003; Feil Seifer and Mataric, 2009; Diehl et al., 2012). Our third working hypothesis (Hypothesis 3) is that in robot-assisted experimental conditions the child produces prosocial behaviors not only toward the robot but also toward humans. For the sake of clarity, a behavior is coined below as “prosocial” only in case it is dedicated to human, not to robot.

### 1.2. Building Up Robot Acceptance

In order to fulfill acceptance criteria of end-users, robot-assisted interventions should meet the efficiency standards of health services, tasked with assessing the level of experimental evidence supporting the added value of newly created tools (Burns et al., 2011), and providing recommendations to practitioners (GRADE Working Group, 2004). To accumulate such supportive evidence, multiple experiences should show that interventions for ASD work better when assisted by robots than in control condition, without the help of electromechanical devices.

To date, such evidence is scarce (Miguel Cruz et al., 2017). Of the 758 studies on robot-assisted interventions for ASD listed by Pennisi et al. (2016), only 29 (0.04%!) were selected as meeting clinical concerns. Publications still too often take the form of pilot studies (e.g., Werry et al., 2001; Miyamoto et al., 2005; Duquette et al., 2008; Robins et al., 2009; Costa et al., 2011; Dickstein-Fischer and Fischer, 2014) without control conditions, inferential statistics, diagnosis methods and inclusion/exclusion criteria (see Pennisi et al., 2016; Ismail et al., 2019 for critical reviews). Although necessary as a starting point, these preliminary studies are unable to establish the effectiveness of robotic tools in clinical samples, according to the rules of clinical methodology (Kazdin, 1998). The best-established effect is the “likability” of robots (Begum et al., 2016): children with ASD show enthusiasm for robotic devices and willingly participate in games assisted by these devices (Pliasa and Fachantidis, 2019)

To fit the needs of special needs educators, a collaborative approach was adopted. The idea of the robot in this project was born in 2011 in France when a father asked a team of young engineers from School of Industrial Biology at Cergy Pontoise to create games for his child with ASD. In 2014, a newly created French start-up created a low-cost, remotely controlled robot ball, that moves by rolling, vibrates and illuminates its transparent cover with different colors. Similar to spherical GIPY-1 (Giannopulu, 2013), Roball (Michaud et al., 2005), or SPRK+ Sphero (Golestan et al., 2017) the robot belongs thus to nonhumanoid devices.

The management team controlling the workflow enrolled the special educators and the children with ASD, and only then tasked researchers who could identify the educational goals and develop the procedure for the robot-assisted psychosocial skills training intervention. Children enrolled displayed low-functioning ASD, that is, intellectual quotient lower than 70 (i.e., intellectual dysfunction). Note however that the focus lies here on the effectiveness of the robot-assisted intervention, not on the specific functioning of these low-functioning children. At the end of our mission, we administered an acceptance questionnaire to analyze whether and how special educators accepted the robot-assisted intervention. We hoped that the intervention is judged as useful and fitting work routines (Hypothesis 4).

### 1.3. Intervention

We proposed an educational intervention targeting social skills and evaluated how efficient the robot is, as compared to the intervention without robotic help. In order to teach the social skills, we designed two sets of lessons to be taught using the *Applied Analysis of Behavior* (ABA) (Cooper et al., 2019) educational method recommended by health services. The key idea of ABA is to increase the probability of desirable behaviors by providing reinforcers in the form of rewards (Skinner, 1981). For the purpose of the present study, we chose the two general social skills that are most often targeted by educational interventions in ASD: requesting and turn-taking (Still et al., 2014; Huijnen et al., 2017). Requesting allows children to initiate a social interaction, express their needs and seek help, and leads to greater independence. Turn-taking is involved in the regulation of any social interaction. In order to exploit the added value of robots, compared with computer-mediated therapy, we administered tasks requiring body displacement in space, in particular during turn-taking lessons.

In line with ABA, the principles of the *Pivotal Training Method* (PTM) (Koegel et al., 1999, 2001) proposes that the learning of general skills (here: turn-taking and requesting) should bring about collateral improvements in a variety of nontrained prosocial behaviors in interpersonal interaction. In the present study, we thus focused on these expected collateral improvements, hoping that nontrained prosocial behaviors (here: orienting toward human, physical contact with human, pointing to communicate enjoyment and interest etc.) are more frequent in the robot-assisted than in control condition (viz. Hypothesis 3).

To sum up, the goal of these analyses was twofold. (1) First, we assessed the efficiency of the robot as a reward deliverer (viz. Hypothesis. 1), as an undesirable behavior reducer (viz. Hypothesis 2) and as social mediator (viz. Hypothesis 3). We expected that positive reactions to reward and nontrained prosocial behaviors are more frequent and that undesirable behaviors are less frequent in the robot, as compared to the control condition. (2) Second, we evaluated the acceptance of robot-assisted intervention by special educators (viz. Hypothesis 4). As in collaborative research interventions are administered by real caregivers, we anticipated that they could derail from the experimental procedure dictated by experimenters (viz. Hypothesis 5).

According to the suggestions of collaborative approach (Dingfelder and Mandell, 2011; Marchand et al., 2011), we conducted our study in two steps. After designing the first set of lessons devoted to requesting, we made successive modifications to the experimental protocol as problems emerged. Only then was the second turn-taking set of lessons administered and used for further analyses.

## 2. Methods

### 2.1. Participants

The teamwork coordinator enrolled 20 children with ASD and 15 special educators in the study. They came from five special-needs schools and centers in France (APEAI Ouest Herault in Béziers, ADAPEI Papillons Blancs in Dunkirk, ADAPEI Papillons Blancs d'Alsace in Mulhouse, Ar'Roch in Rennes, DASCA Adéle de Glaubitz in Strasbourg, ADAPEI 44 in Nantes, APPARTE) where children receive care for their behavioral disorders.

As these centers correspond to small structures taking care of children with various mental disorders, only 1–2 individuals in each center fitted our inclusion/exclusion criteria: (1) 60–122 months of age at enrollment; (2) developmental age of 18–30 months assessed by verbal and preverbal cognition subtest from Psychoeducational Profile (PEP-3) (Schopler et al., 2004) (see below); (2) a diagnostic of ASD made by expert psychologists from Regional Autism Resources Center, and reconfirmed here by *Social communication Questionnaire* (SCQ, Rutter et al., 2003, see below); (3) no identifiable neurological disease or major neurological treatment. The ratio between developmental and maturational age was 0.28 (*SD* = 0.09), qualifying the children as low-functioning (i.e., severe intellectual deficit). Further psychological characteristics of our sample are provided in Table 1. The female-male ratio was 3/17.

**Table 1:** Psychological tests used in the present experiment.

**Test**	**No. items**	**Item scoring**	**Score interpretations**	**Child score**
SCQ	40	0–1	score>15: possible ASD	21.43 (3.06)
V-listening	20	0–2	score <70: delay in receptive communication	16.81 (9.62)
V-speaking	32	0–2	Score <70: delay in expressive communication	22.15 (7.95)
V-autonomy	27	0–2	score <70: delay in personal autonomy	39.96 (15.46)
V-socialization	26	0–2	score <70: delay in socialization	22.08 (7.09)
V-adaptation	30	0–2	score <70: delay in social adaptation	9.56 (6.85)
PEP-3: AEs	11	0–2	Higher score: better affective expression	9.93 (4.43)
PEP3: SR	11	0–2	Higher score: better social reciprocity	11.14 (4.02)
PEP3: CVPV	34	0–2	Provides developmental age	26.44 (7.37)
SPCR	85	0–1	Higher score: more sensory abnormalities	26.86 (5.64)
ESES	13	1–9	Higher score: higher self-efficacy belief	85.86 (10.31)

*For each test, the number of items, score range, score interpretation, mean score and standard deviation (SD) are provided for the children with ASD. SCQ, Social Communication Questionnaire; PEP, Psychoeducational Profile; SPCR, Sensory Profile Checklist Revised; AE, affective expressions; SR, social reciprocity; V, Vineland*.

As the robot had a low level of autonomy (Level 2; see Parasuraman et al., 2000), in each experimental session, in addition to the special educator interacting with the child, another person controlled the robot. Fifteen educators who cared for the children applied the experimental protocol: 56% were special needs monitors and 31% were special needs professionals, 67% had more than 10 years of experience, and 93% were women. At least one special educator in each center reported having already undergone a short ABA familiarization course. Just under half (47%) stated that they had never used new technologies, and just over half (53%) that they used them occasionally. The interventionist and the families of all the children received a letter explaining the goals, experimental procedure and rights of parents and children, and provided their written informed consent, in accordance with the Declaration of Helsinki. Each parent completed a form provided by the University of Toulouse informing them about their rights and predictable risks in comparison with foreseeable benefits. An ethics and scientific committee of the consulting company in the role of intermediary between the start-up, researchers and investors approved the experimental protocol; the committee members were also present during the first meeting. A declaration of ethical collection and storage of data was also made to the French Data Protection Authority (CNIL; ref.: 7e42415863j).

### 2.2. Material

#### 2.2.1. Robot

We used a white, spherical prototype, measuring 18 cm in diameter and weighing 900 grams that was enclosed in a transparent plexiglass sphere resistant to shocks and pressure. Designed with a smiling face, equipped with actuators (LEDs, motors) and sensors (IMU 6-Axis, RFID), the prototype could light up or blink in different colors, and moved on two wheels in contact with the sphere. The robot was powered by AAA batteries and had autonomy of 3 to 4 h. Its behavior was remotely controlled by a touch pad (iPad iOS 10 or 11) with which it communicated through Bluetooth Low Energy over a distance of about 20 m.

In view of the intervention, three key functions were programmed in the robot. It acted as reward deliver, displaying colored lights and spinning movements, and also as cue provider: it offered specific lights and displacements prescribing required behavior of the child (e.g., “Touch the robot if it is your turn and if the robot is lit up in blue,” see **Table 5**). Finally, it acted as lessons organizer, as explained below.

Two sets of seven lessons were developed. The application on the graphic tablet allowed the interventionist to consult the child's profile, which contained his/her experimental history and preferred sensory rewards, select a lesson, and display the lesson description and lesson control panel. The control panel featured various icons to launch the robot's cue, record the child's response, and provide rewards. Four types of responses from the child could be recorded: failure, success with total prompt, success with partial prompt, and success without prompt.

The control programs were developed on C++ for the robot and on Swift for the tablet. We were not allowed by investors to provide more technological details or the name of the device, never described in the literature and not commercialized to date.

In addition to the robot, a shoulder strap was provided to hold the graphic tablet. For the purpose of the experiment, a GoPro camera (Hero), a tripod (Fotopro), and a memory card (microSDHC SanDisk Extreme 32) were given to each center. A child's chair, and hoops were also required for the intervention. Because of the spherical design of the robot, balls were used in control condition.

#### 2.2.2. Tutorials

Three tutorials were offered to the educators: (1) a brief introduction to ABA; (2) a technical description of the robot (see section 2.2.1), together with a detailed presentation of each set of lessons (see section 2.2.3); and (3) a description of the experimental design underlying the intervention (see section 2.3.2).

**Figure 1:**
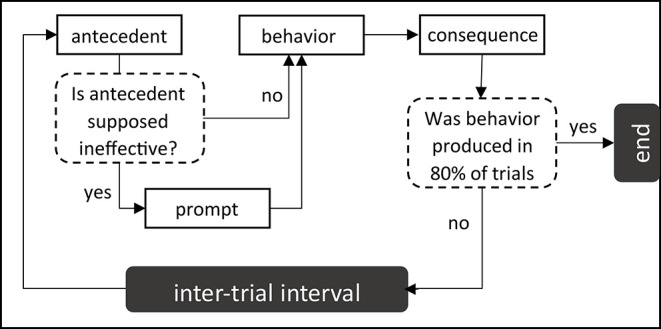
ABA-based procedure of learning.

In the description of ABA, we recalled that in line with the principle of selection by consequences (Skinner, 1981), the educators would have to manage the sequence of events controlling each child's behavior (antecedent, behavior, consequence). According to the Discrete Trial Training method (Smith, 2001) learning should take the form of trials, each sequence involving an antecedent cue anticipating the appropriate behavior (e.g., “Touch the robot in turn”), a prompt wherein the educator assists the child (e.g., demonstration of required gesture, hand-over-hand assistance, pointing, nodding etc.), the child behavior (e.g., touching the robot in turn), the environmental consequence (e.g., verbal praise), and the intertrial interval (see Figure 1). We explained that providing a reward immediately after the to-be-learned target response reinforces the latter, increasing the probability of the target response being produced in the future.

Before each session, given their knowledge of the child's abilities and needs, the educators were asked to anticipate the required level of prompting, to avoid delays between the instruction and the prompt. They were told they should not hesitate to start with all prompts to facilitate learning. Prompts should be gradually faded out as learning proceeded, or increased in the case of a child failing (Leaf et al., 2016). We explained how instructions and rewards should be efficiently applied (e.g., brief, clear, short and consistent instructions, provided when the target response was not being produced; reward applied immediately after the target response). Undesirable behaviors had to be gently and briefly interrupted, and the child immediately prompted to provide the target response (Cividini-Motta et al., 2019).

The descriptions of each lesson given to educators interacting with the child contained the learning goal (e.g., “Touch the robot in turn”), corresponding verbal instruction (e.g., “It's your turn”), required material (e.g., child's chair), preparation procedure (e.g., place the robot in the center of the room), a step-by-step procedure for learning, and a validation criterion (see below).

#### 2.2.3. Sets of Lessons

Given that the volume of the tutorial depicting the lessons was 30 pages long, we provide below an abbreviate illustration of its content. Each set comprised a learning procedure that was ultimately aimed at enabling children to produce spontaneously and appropriately the general social skill targeted by the intervention: requesting (Set 1) and turn-taking (Set 2). Each set was composed of seven lessons, each with a learning goal, corresponding to a *required response* to be acquired by the child (e.g., “Look ate the robot,” see Table 2, or “Touch the robot in turn”, see Table 3). Required responses progressed from simple to complex, from prompted by the educator to initiated spontaneously by the child, from centered on the toy (robot or ball) to centered on the interaction with the educator (see Tables 2, 3 for the sequence of lessons in each set).

**Table 2:** Required responses (R) for requesting set of lessons.

**Set 1**	**Requesting**
R1.	Look at the robot
R2.	Get closer to the robot
R3.	Touch the robot
R4.	Get closer to and touch the robot
R5.	Hold inactive robot to the adult
R6.	Hold inactive robot to the adult, who then activates it
R7.	Spontaneously hold inactive robot to the adult, who activates it

**Table 3:** Required responses (R) for turn-taking set of lessons.

**Set 2**	**Turn-taking**
R1.	Touch the robot in turn
R2.	Touch the robot if it is your turn and if the robot is lit up in blue
R3.	Get closer to and touch the robot, in turn
R4.	If it is your turn and if the robot is lit up in blue, get closer and touch the robot
R5.	If it is your turn and if the robot is lit up in blue, imitate the adult who followed the robot along a short distance
R6.	Wait until the robot has reached the end of a short pathway and, if it is your turn and if the robot is lit up in blue, follow the path and touch the robot
R7.	Touch the robot to select the color controlling the turn-taking; wait until the robot has reached the end of a short pathway and, if the robot is lit up in blue, follow the path and touch the robot

In each lesson, a step-by-step procedure described the elementary actions required from the robot (e.g., light up in blue), the interventionist (e.g., say “It's my turn”), and the child (e.g., “Touch the robot in turn”). Each lesson entailed five discrete learning trials (e.g., five turn-takings) where the interventionist attempted to elicit the required response. In accordance with ABA criteria, a required response was deemed to be acquired if it was produced in 80% of these trials, without or with partial prompting (see Figure 1). If, after the five repetitions of the same trial, the child failed to meet this criterion, the educator stopped the whole experimental protocol. The step-by-step procedure for the first lesson in the turn-taking set appears in Table 4, for the second lesson in Table 5.

**Table 4:** Step by step procedure for the first lesson in the turn-taking set.

**Required response**	**Touch the robot in turn**
Antecedent	1. Sit facing the child, and place the robot between you. The robot is inactive.
	2. Touch the robot on the top: it will light up in blue for a moment.
	3. Then encourage the child to do the same. Each time, say “It's my turn / It's your turn.”
Behavior	4. If the child respects his/her turn, the robot will light up in blue.
	5. If not, the interventionist will blocks him/her, saying “No, it's my turn”.
	6. If the child does not attempt to touch the robot, the educator selects a guidance specific to the child.
Consequence	7. After an errorless sequence of six turn-takings, the robot provides a sensory reward (specific to each child) and the interventionist gives verbal praise.
Validation Criterion	8. Repeat the sequence of turn-takings 5 times in a row (30 trials in all).
	9. Go to the next lesson if the child has produced a correct turn-taking sequence four times out of five.

**Table 5:** Step by step procedure for the second lesson in the turn-taking set.

**Required response**	**Touch the robot if it is your turn and if the robot is lit up in blue**
Antecedent	1. Sit facing the child, and place the robot between you. The robot is active and lit up either in blue or red.
	2. If the robot's light is blue say “The robot is blue! Touch it!.
	3. If the robot's light is red say “The robot is red! Don't touch it!”.
Behavior	4. If the robot's light is red and the child reaches to touch it, the educator will block the gesture, saying “The robot is red! Don't touch it!”
	5. If the robot's light is blue and the child does not attempt to touch it, the educator selects a guidance specific to the child (ex. The light is blue, you can touch it).
	6. If the robot lights up in blue and the child touches it, the robot light up in white for a moment.
Consequence	6. After an errorless sequence of six turn-takings, the robot provides a sensory reward (specific to each child) and the educator gives verbal praise.
Validation Criterion	7. Repeat the sequence of turn-takings 5 times in a row (30 trials in all)
	Go to the next lesson if the child has produced a correct turn-taking sequence four times out of five.

#### 2.2.4. Workbooks

Information about the children and their caregivers was collected in two workbooks. The first workbook collected general information about the child (i.e., age, diagnostic tools used, developmental age) and provided five psychological tests for psychometric assessment: *Social Communication Questionnaire* (SCQ) (Rutter et al., 2003), *Vineland II* (Sparrow et al., 2012), *Psychoeducational Profile* (PEP-3) (Schopler et al., 2004), *Sensory Profile Checklist Revised* (SPCR) (Bogdashina, 2012), and *Educators' Sense of Efficacity Scale* (ESES), adapted from Teachers' Sense of Efficacity Scale (Tschannen-Moran and Hoy, 2001). These tools are described in Appendix 1; their key features and interpretation in Table 1. The second workbook included Educators' Sense of Efficacity Scale and the acceptance questionnaire.

#### 2.2.5. Post-intervention Acceptance Questionnaire

To assess acceptance of the intervention, we developed a questionnaire for the educators targeting several issues: (1) for what kind of children is a robot-assisted intervention best suited? (2) what is its added value, advantages and disadvantages? (3) what is its effect on workload, educational intervention, and children's learning? and (4) what training is required to use the robot in educative intervention?

### 2.3. Procedure

#### 2.3.1. Collaboration Procedure

In the present work, the stakeholders first met in order to discuss ethical issues, methodological requirements, and acceptance of the intervention by the children and educators. Two training meetings were organized for them. In the first training meeting, held before the start of experimentation, researchers described the experimental goals and procedure, simulated learning sessions, and described how to manage challenging behaviors. The second training meeting took place during the administration of the first set of lessons: the experimenters provided feedback to the educators, using videos of previous learning sessions. Half a day each week, a hotline was manned by JK to answer the educators' questions. The final meeting took place after the experimentation, in order to present the results and discuss the strengths and weaknesses of the robot-assisted intervention. Each family received a brief summary of their child's progress.

#### 2.3.2. Intervention Procedure

**Figure 2:**
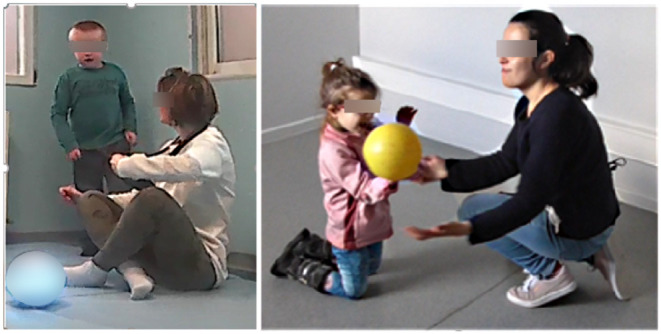
Intervention conditions: with robot **(left)** and with ball **(right)**.

After the educators had taken notice of ABA principles, of lessons content, and of the experimental design, described in the tutorials (see section 2.2.2), they completed the psychological tests from the first workbook (see Table 1). Then, the children underwent a familiarization session, where they were merely put in the presence of an inactive robot. The following week, the lessons started: requesting (see Table 2) followed by turn-taking (see Table 3), according to the step-by-step procedure as described in the tutorials (see Tables 4, 5). Each child was administered each lesson in two conditions, in random order: with the robot and with the ball (see Figure 2). At least one session with the robot and one with the ball was administered for each lesson. Each set of lessons was taught over 12 weeks. The entire intervention took place over 24 weeks. After the intervention, a second workbook was provided, including the ESES and acceptance questionnaire.

### 2.4. Data Reduction and Analysis

#### 2.4.1. Observation Grid

After the end of interventions, the method of direct observation from videos was used (Hops et al., 1995). Video recordings of all the experimental sessions were analyzed by two trained coders (psychology undergraduates), who were familiar with ABA and blind to the purpose of the experiment. They used an observation grid listing 16 categories of responses (e.g., proximal pointing, head/gaze oriented toward human, stereotypies, see Table 6, right column), organized in four global classes: positive reactions to reward, prosocial behaviors, undesirable behaviors, and orientations (see Table 6, left column). To assess child autonomy, coders were required to record the prompts initiated by educators. To assess implementation quality, they were also asked to record the educators' implementation errors. Cohen's kappa was calculated to measure interrater agreement (*k* = 0.92).

**Table 6:** Dependent variables and to-be-observed response catégories.

**Dependent variables**	**Response categories**	**Label**
Positive reactions to reward	To reward delivered by human	(PRH)
	To reward delivered by robot	(PRR)
Prosocial behaviors	Proximal pointing	(PP)
	Distal pointing	(DP)
	Joint gazing	(JG)
	Physical contact with human	(CH)
	Head/gaze oriented toward human	(OH)
	Social smiles	(SS)
	Desirable vocalizations	(DV)
Orientations	Head/gaze targeting human	(OTH)
	Head/gaze targeting toy: ball or robot	(OTT)
Undesirable behaviors	Inappropriate behaviors	(IA)
	Stereotypies	(S)
	Undesirable vocalizations	(UV)
	Lack of interest	(LI)
	Attentional dropout	(AD)

*Each dependent variable in the left hand column is a combination of responses categories shown in the middle column. Left hand colums displays response category labels*.

#### 2.4.2. Dependent Variables

All dependent variables were measured after the end of interventions. For each child and each experimental condition (robot, ball), we recorded the number of times each response category (e.g., proximal pointing) occurred, resulting in 16 summed scores (see Table 6, right column). These scores were then combined to four dependent variables corresponding to above-mentioned global classes (i.e., positive reactions to reward, prosocial behaviors, undesirable behaviors, and orientations, see Table 6, left column).

To take a deeper look into the effect of robot-assisted intervention, we computed the proportion of prosocial and undesirable behaviors produced in robot condition. The proportion was then normalized (from 1 to −1):

(1) $$\begin{array}{l} Normalized.Proportion=2\times \left(\frac{x_{robot}}{x_{robot}+x_{ball}}\right)-1 \end{array}$$

The normalized proportion takes a positive value when most of these behaviors were produced in robot condition, and inversely:

(2) $$\begin{array}{l} \{\begin{array}{c} 1\;if\;x_{robot}>x_{ball}\\ 0\;if\;x_{robot}=x_{ball}\\ -1\;if\;x_{robot}<x_{ball} \end{array} \end{array}$$

In the formula, x_*robot*_ and x_*ball*_ refer to the number of behaviors produced in robot and ball condition, respectively.

#### 2.4.3. Statistical Analyses

To capture the characteristics of the children for whom the intervention was stopped and those who passed from lesson to lesson, one-tailed *t*-tests were carried out on all psychological test scores. Three groups were compared: the group who stopped the first set of lessons (i.e., Requesting), the group who started the second set (i.e., Turn-taking), and the group who completed the second lesson of the second set.

For further analysis, four experimental factors were envisioned: Condition (robot, ball), Reaction target (human, toy), Orientation Target (human, toy), and Prompt (with, without). Note, for Reaction target and Orientation target, the toy refers to robot in robot condition and to ball in ball condition.

To assess the efficacy of the robot-assisted intervention, we ran three statistical analyses. A 2 (Reaction Target = human in robot condition, human in ball condition, robot in robot condition) ANOVA was performed on positive reaction to reward and a 2 (Orientation Target = human, toy) × 2 (Condition = robot, ball) ANOVA was on orientations. A 2 (Condition = robot, ball) × 2 (Prompt = with, without) ANOVA was also run on prosocial behaviors and on undesirable behaviors to check whether the robot improved the children's social skills.

In all the ANOVAs, repeated measures were used on all dependent variables. Because each experimental factor (Condition, Reaction Target, Orientation Target and Prompt) had two levels, the assumptions of sphericity and of homogeneity of variances were always met. The distributions of dependent variables did not diverge from normal, as indicated by Lilliefors test for normality (*D* = 0.1052, *p* = 0.5939; *D* = 0.0636, *p* = 0.99; *D* = 0.1145, *p* = 0.2722; *D* = 0.0443, *p* = 0.9901, for reactions to reward, prosocial behaviors, orientations and undesirable behaviors, respectively).

If required, the ANOVAs were followed by appropriate two-tailed *t*-tests. The sign of normalized proportion was tested using one-sample *t*-test with 0 as comparison value. Finally, a matrix of correlation indices (r) was computed using all scores from the psychological tests and categories of responses. For all the above-mentioned analyses, the significance level was set at *p* <0.05, with the corresponding estimates of the effect size (η^2^).

#### 2.4.4. Statistical Analyses for Single Participant

Single-participant analyses were then performed on one of the children with ASD who successfully completed the whole intervention protocol. For this dataset, Bayesian statistics for single cases (de Vries and Morey, 2013; de Vries et al., 2015) were used. The posterior distribution for the standardized mean differences and Bayes factors were computed using the JZS+AR model with 10,000 Gibbs sampler iterations (de Vries et al., 2015). The Bayes factor quantifies evidence in the data for the null hypothesis against the alternative one: an inverse Bayes factor (1/BF) greater than 1 supports the alternative hypothesis. All 16 categories of responses, together with prosocial behaviors and undesirable behaviors, were submitted to this analysis.

#### 2.4.5. Descriptive Statistics

To provide a glimpse into implementation fidelity, that is, the degree to which the educators strayed from the procedure specified by the experimenters, the coders were required to record any implementation error. The frequency of the failures was computed as a ratio of the number of failures to the number of videos. Finally, responses to the acceptance questionnaire were scored as percentages.

## 3. Results

### 3.1. Child Sample Results

Children's Vineland-II and PEP-3 scores in our sample were low (see Table 1), indicating severe delays in social adaptive behavior, as well as in AE and SR skills. On average, sensory abnormalities were moderate. Of the 20 children with ASD who were initially enrolled, 15 reached the second set of lessons. The five participants who had to stop the first set had lower Vineland scores on listening, speaking and autonomy than the remaining participants, *t*_(18)_ = 3.20, *p* <0.007; *t*_(18)_ = 3.04, *p* <0.007; and *t*_(18)_ = 2.29, *p* <0.032. Of the 15 children who started the second set of lessons, only eight completed it. These eight children had higher Vineland listening scores than those who failed to complete the first and second sets of lessons, *t*_(13)_ = 2.23, *p* <0.044.

### 3.2. Robot-Assisted Intervention Results

#### 3.2.1. Reward Deliver

**Figure 3:**
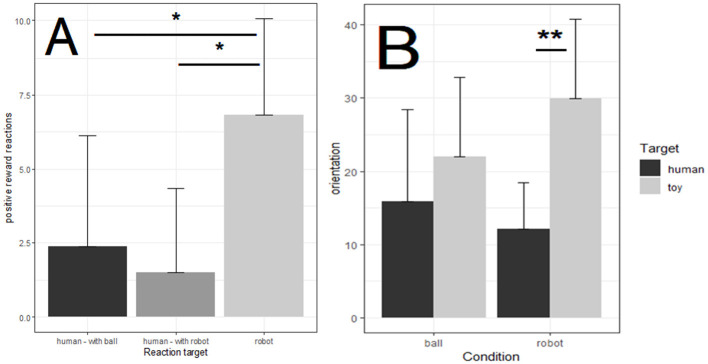
Positive reactions **(A)** and orientations **(B)** as a function of Condition (ball, robot). *p < 0.05, **p < 0.01and ***p < 0.001.

A 2 (Reaction Target = human in robot condition, human in ball condition, robot in robot condition) ANOVA on positive reactions showed a main effect of Reaction Target, *F*_(2,14)_ = 4.06, *p* = 0.04, η^2^ = 0.546. Corrected pairwise comparisons (see Figure 3A) showed that there was more positive reactions to the reward when it was delivered by the robot rather than by the human in robot and in ball conditions [*t*_(7)_ = 2.37, *p* = 0.049; *t*_(7)_ = 2.50 *p* = 0.04].

#### 3.2.2. Undesirable Behavior Reducer

A 2 (Condition) ×2 (Prompt) ANOVA on undesirable behaviors revealed no statistically reliable effects.

#### 3.2.3. Social Mediator

A 2 (Orientation Target) × 2 (Condition) ANOVA on orientation indicated an important main effect of Orientation Target, *F*_(1, 7)_ = 23.538, *p* <0.002, η^2^ = 0.771. Children oriented more frequently toward the toy (i.e., ball or robot) than toward the educator. As illustrated in Figure 3B, there was also an Target Orientation × Condition interaction, *F*_(1, 7)_ = 12.850, *p* <0.009, η^2^ = 0.647. When the children played with the robot, they oriented more often toward the robot than toward the educator, *t*_(7)_ = 7.78 *p* <0.0001. When they played with the ball, there was no effect of Orientation target, *t*_(7)_ = 1.80, *p* = 0.1142.

A 2 (Condition)× 2 (Prompt) on prosocial behaviors revealed a main effect for Prompt on prosocial behaviors only, *F*_(1, 7)_ = 9.688, *p* <0.017, η^2^ = 0.581: Prosocial behaviors occurred more frequently with the prompt (20.06, *SD* = 10.75) than without it (9.50, *SD* = 8.45).

The value of the normalized proportion of prosocial behaviors was significantly negative, *t*_(6)_ = 2.948, *p* = 0.026: There were more prosocial behaviors in the ball rather than in the robot condition.

#### 3.2.4. ASD Children Characteristics

There was a positive correlation between SCQ scores and orientations toward the ball condition (*r* = 0.794, *p* = 0.033), and a negative correlation between orientations toward the robot and auditory sensory abnormalities (*r* = −0.907, *p* = 0.005).

Further conclusions were drawn from the correlation between the normalized proportion of prosocial behaviors and SCQ score: the more severe the symptoms (i.e., the higher the SCQ value), the lower the proportion of prosocial behaviors produced in the robot as compared to the ball condition (*r* = −0.813, *p* = 0.026).

#### 3.2.5. 6 Longitudinal Single-Participant Analysis

**Figure 4:**
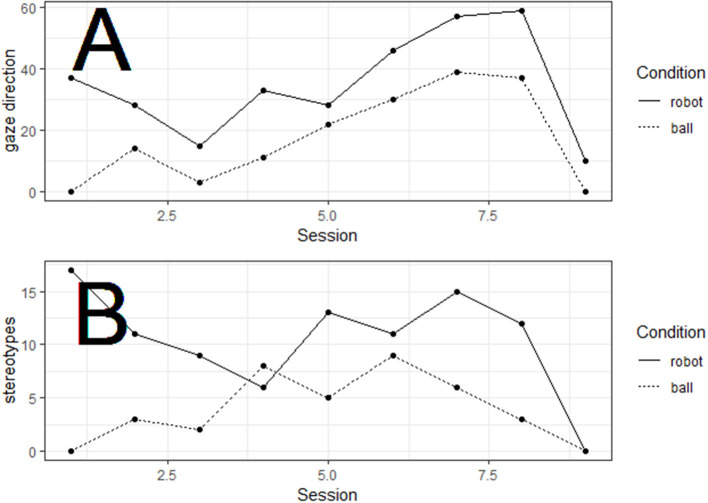
Gaze direction **(A)** and stereotyped behaviors **(B)** as a function of Lessons and Condition (robot, ball).

The child with ASD who completed all the sessions directed his gaze more often toward the robot than toward the ball (1/BF = 1.32 >1) (Figure 4A). He also produced more stereotypic behaviors in the robot than in the ball condition (1/BF = 2.82 >1) (Figure 4B).

### 3.3. Implementation Issues

Given that in collaborative/applied research, experimenters do not have total control of the implementation process and context of the experimental procedure, it is essential to describe the context delivery and the real-world difficulties encountered. This may prove to be particularly valuable in future efforts predicting, avoiding or better adapting to these socio-ecological constraints.

During the first set of lessons, the experimenters and coders identified five implementation failures where educators strayed from experimental requirements: instruction repeated too often or delivered at an inappropriate time; errors in action sequencing (i.e., instruction + prompt + interval, behavior + reward); reward omitted or delivered at an inappropriate time (e.g., before the child's behavior or after a failure); trial omission; and distractors not removed. In the set of 32 videos that were examined, 48 implementation failures were recorded, thus resulting in 1.5 failures per session. As indicated in Table 7, the most frequent failures were associated with reward or trial omission. However, the most severe procedural error was the omission of baseline conditions: before the intervention, the researchers had asked the educators to perform two baseline sessions: one with the robot and one with the ball, but some educators only carried out the baseline condition with the robot.

**Table 7:** Implementation failures.

**Nature of implementation failure**	**Failure frequency**
Instruction	0.16
Action sequencing	0.13
Reward	0.69
Trial	0.47
Distractor	0.06

## 3.4. Robot Acceptance

### 3.4.1. Acceptance Questionnaire

The distributions of responses to the acceptance questionnaire showed that 87% of educators were satisfied or quite satisfied with their experience with the robot, 73% agreed that the robot brought substantial added value and transformed their practice, and 87% wanted to keep on using the robot in the future. Nevertheless, 67% of respondents confessed that they had been tempted to stop the intervention procedure. The reasons they gave included technical (80%) and organizational (33%) difficulties. The list of robot disadvantages also included substantial personal investment (60%) and increased workload (43%). In response to the questions assessing their training requirements, 40% of interventionists deemed that they need training in applying a structured educational approach.

### 3.4.2. Interventionists' Self-Efficacy Assessment

The interventionists' feeling of self-efficacy was initially high, and rose from 78.43 (*SD* = 11.97) before the intervention to 93.26 (*SD* = 10.29) after the intervention on a scale of 0–100, representing a significant increase, *t*_(6)_ = −4.5962, *p* <0.004.

## 4. Discussion

To better understand how to construct robotic tools for individuals with ASD, we conducted a collaborative study assessing the effects of a robot-assisted intervention on children with low-functioning ASD. Our intervention provided mixed results. As expected, children reacted more positive affect to rewards in robot as compared to control condition (viz. Hypothesis 1), and educators were quite enthusiastic about the robotic help in the learning task (viz. Hypothesis 4). However, contrary to our expectations, our robot was not able to act as a social mediator (viz. Hypothesis 3): when children played with the robot, they payed more attention to the toy than to the educator and the proportion of prosocial behaviors was higher in the control condition. Undesirable behaviors did not decrease (viz. Hypothesis 2). Of interest, the progression in the curriculum was IQ-specific: among the children we enrolled, those who displayed higher listening skills moved easily from lesson to lesson.

### 4.1. Reward Deliver

Children with ASD had more positive reactions to reward delivered by robot rather than to praises delivered by the eductor. This observation is analogous to enthusiastic reactions to robot reported in previous case studies (Dautenhahn, 1999, 2000; Kozima et al., 2007).

This enhanced reaction did not generalize to rewards delivered by the educator in robot condition though. The robot did not act as a general motivator (i.e., “motivating operation,” Laraway et al., 2003; Edwards et al., 2019) enhancing the reinforcing effectiveness of any reward delivered in its presence. Rather, it acted as a preferred object: a strongly attractive object for children with ASD (DeLeon et al., 2001). In further studies, robots might be thus used to reinforce behaviors targeted by interventions, and compared to already exiting preferred toys.

### 4.2. Undesirable Behavior Reducer

Our robot had no consistant effect on undesirable behaviors: stereotypic behaviors even increased in one child. Ismail et al. (2012) suggested that robots may contribute to reduce the frequency of stereotypic behavior only for children with mild or no intellectual deficit. This demonstrates the need for psychometric descriptions of children in studies on robot-assisted interventions.

### 4.3. Social Mediator

The proportion of prosocial behaviors was higher in the control condition, rather than the robot-assisted intervention. We failed to offer support to social mediator hypothesis. Robins et al. (2005) warned that instead of social mediator, robots may sometimes take the role of social isolator. Meucci et al. (2019) suggested that the advantage of the interaction with a robot depends on the level of intellectual functioning of the children with ASD. In our data, we indeed noted that the more severe the ASD the lower the proportion of prosocial behaviors produced in the robot condition.

Note, extant information on social mediator hypothesis mostly comes from pilot studies or technical reports, without control condition, descriptive and inferential statistics (Werry et al., 2001; Robins et al., 2009; Iacono et al., 2011; Shamsuddin et al., 2012) and/or without diagnostic method, exclusion and inclusion criteria, developmental age etc. (Feil Seifer and Mataric, 2009; Valadao et al., 2016). Further studies could better comply with the requirements of clinical methodology.

Our intuition here is that using a highly attractive tool comes with the risk of turning the child with ASD away from the interpersonal social interaction skill, target of the training program. Our data indeed showed that children with ASD primarily gazed at the toy, seeing it as more attractive than the educator, in line with Social Motivation Theory of Autism (Chevallier et al., 2012; Delmonte et al., 2012). We suppose that robots would be more likely to “catalyze” prosocial behaviors if they interacted directly with the child, without any remote control, and if they endorsed a social role: that of prompter, teacher, helper in critical situations, etc. (Zubrycki and Granosik, 2016; Huijnen et al., 2017). Children with ASD would be therefore efficiently trained to produce and interpret social cues exchanged with the robot, and perhaps could generalize this learning to interpersonal interaction. In future research, robots of higher autonomy, similar to Jibo (Guizzo, 2015) or MINA (Ghorbandaei Pour et al., 2018) deserve particular attention.

### 4.4. Sensory Aversions and Inter-individual Heterogeneity Issue

Before the intervention, we feared that our robot, with its lighting signals and noisy functioning, might trigger anxiety among the children with ASD. The Intense World Theory of Autism (Markram, 2007) warned us indeed that children with ASD may be hypersensitive to these stimuli. This turned out to be a legitimate concern, as most of the auditory-sensitive children turned away from the robot.

This finding underscores the overlooked challenge faced by robots in the context of ASD: the inter-individual heterogeneity of children with ASD is shaping their reactions. This inter-individual heterogeneity makes it unlikely that a given robot or a given intervention will work for all children with ASD. In clinical settings, interventionists are used to adjust to each individual (Stahmer et al., 2011). They identify the sensory and cognitive particularities of each individual in order to decide which toy and which educational goal may be selected. They determine in real time how to attract the child's attention and modulate child anxiety, and which instructions, prompts, rewards and pauses should be administered. In further studies, robots should be endowed with an extensive set of educational goals and sensory options so that the administration of the educational procedure can be personalized. A first step toward this goal was recently made by Clabaugh et al. (2019) who developed a fully autonomous robot, SPRITE, able to personalize its instruction and feedback to each child's proficiency.

### 4.5. Collaboration Issues

One of the most often debated issues in the field of robotic assistance for children with ASD is infringement of the methodological rules of clinical research (Kim et al., 2012; Pennisi et al., 2016). This was an acute problem in our participatory study too. In the face of the understandable enthusiasm of the other stakeholders, it was difficult for the researchers to make their warnings heard. Nonexperimentalists have difficulty accepting that the violation of methodological rules inexorably means that some of the data that are collected are unusable.

Despite the obvious advantages of participatory research, it is important to acknowledge that this strategy creates huge problems in terms of coping with the priorities and constraints of different stakeholders, often working at cross purposes (Kim et al., 2012). Evidently, investors need to deliver a compelling marketable innovation capable of a sustainable commercial growth. Engineers want to promote innovative technological platforms that make existing ones obsolescent (Kim et al., 2012). Researchers are concerned with the originality and efficacy of the educational intervention, and thus need to respect to rigorous methodological criteria (Pennisi et al., 2016). The special need educators are interested in creating a user-friendly, personalizable tool that meets the specific needs of individual patients and fits in with current learning routines (Boardman et al., 2005). The company organizing the project has to factor in the time-limited and evanescent nature of the funding. There may be insufficient time and financial resources to organize meetings in order to build communication and trust between partners and work out a consensus on the standards of excellence to be met.

### 4.6. Implementation Fidelity Issue

As feared, the educators derailed from procedure dictated by research design (viz. Hypothesis 5). Despite workbooks, demonstrations and a hotline, educators made 1.5 implementation failures per session. In this respect, our intervention attempt was no different from others: Stahmer et al. (2015). showed that even after 28 h of intensive workshops, followed by 2 years of observation and coaching, the percentage of sessions meeting 80% implementation fidelity was just 60% for discrete trial teaching and as low as 20% for pivotal response training. Contrary to academic staff, special needs educators do not undergo years of training in administering trial-based, experiment-like procedures. Their skills imply intimate understanding of the child's difficulties and needs. Our intuition is that robots may play a non-negligible role here. If they can be designed to free educators from structuring the intervention according to the guidelines of educational protocol, they may contribute to the dissemination and application of structured educational approaches (e.g., ABA) recommended by health services.

### 4.7. Acceptance of the Robot-Assisted Intervention

The educators who took part in the present study were highly satisfied with their interaction with the robot. Coders noted that they seemed to take greater pleasure in interacting with the children. They had a greater feeling of self-efficiency after the experiment. Although we suspect that responses to the self-efficiency questionnaire were affected by a social desirability bias (Troye and Supphellen, 2012), leading the care staff to ignore undesirable traits such as self-doubt, it is quite possible that being supported by a robotic tool, instead of facing the child alone, engendered feelings of relief and satisfaction.

## 5. Conclusion

To better understand how to construct convincing tools for individuals with ASD, we conducted a collaborative study that assessed the effects of a robot-assisted intervention on both the prosocial and undesirable behaviors of children with low-functioning ASD. The robot attracted orienting responses from the children and the rewards it offered elicited more positive responses, but it failed to act as a social mediator: it did not motivate desired social behaviors toward humans. Robotic assistance was obviously judged to be positive by educators, thus contributing to the dissemination of evidence-based practices for individuals with ASD. In further studies, robots with higher levels of autonomy and differentiation, of richer set of educational goals and sensory response options might be tested as reinforcers of social behaviors targeted by educative intervention.

## Data Availability Statement

The raw data supporting the conclusions of this article can be found through Figshare (10.6084/m9.figshare.11994801).

## Ethics Statement

The studies involving human participants were reviewed and approved by the Research Ethics Committee for Non-Invasive Procedures (CERNI) of the Université Fédérale Toulouse Midi-Pyrénées. The interventionist and the families of all the children received a letter explaining the goals, experimental procedure and rights of parents and children, and provided their written informed consent, in accordance with the Declaration of Helsinki. Each parent completed a form provided by the University of Toulouse informing them about their rights and predictable risks in comparison with foreseeable benefits. Written informed consent was obtained from the individual(s) or the individuals' legal guardian/next of kin for the publication of any potentially identifiable images or data included in this article.

## Author Contributions

JK designed the study and supervised data collection. VK analyzed the results and wrote the manuscript. JK and VK critically reviewed and edited the manuscript for important intellectual content. All authors approved the final manuscript.

### Conflict of Interest

The authors declare that this study received funding from AG2R La Mondiale. The funder was not involved in the study design, collection, analysis, interpretation of data, the writing of this article or the decision to submit it for publication.

## Appendix 1

### List of Psychological Tests Used

The *SCQ* is a screening tool based on the DSM-IV criteria for autism and the *ADI-R* algorithm (Rutter et al., 2003). It takes the form of a standardized parent questionnaire to assist in autism diagnosis by capturing key autistic symptoms (e.g., “did he/she ever show you things that interested him/her to engage your attention?”).

The *Vineland-II* is a structured interview administered to primary caregiver(s) to assess a child's daily living skills (e.g., “looks at the caregiver when s/he hears his voice”). Using this tool, we evaluated three domains (communication, daily living skills, socialization), thus obtaining an overall adaptive behavior evaluation.

The *PEP-3* identifies learning strengths and facilitates the selection of educational programs for children with ASD. In the present study, we scrutinized affective expressions (AE; e.g., “manifests an appropriate level of fear”) and social reciprocity (SR; e.g., “initiates social interactions”).

The *SPCR* assesses unusual sensory experiences of individuals with ASD (e.g., “covers ears when hears certain sounds”).

The *ESES* comprises 13 items evaluating interventionists' beliefs about their efficiency in controlling children (e.g., “I am able to copy with disruptive behavior in a teaching session”).

The *SCQ* and *Vineland-II* yield standardized scores, while the others yield raw scores. A high *SCQ* score indicates a severe form of ASD-like symptoms. For the remaining tests, low scores indicate a severe functional impairment (see Table 1).
